# A review of seizures and epilepsy following traumatic brain injury

**DOI:** 10.1007/s00415-020-09926-w

**Published:** 2020-05-22

**Authors:** Surina Fordington, Mark Manford

**Affiliations:** 1 University of Cambridge, Cambridge, UK; 2 Department of Clinical Neuroscience, University of Cambridge, Cambridge, UK

**Keywords:** Epilepsy, Traumatic brain injury, Dissociative seizures

## Abstract

Traumatic brain injury (TBI) is one of the commonest presentations to emergency departments and is associated with seizures carrying different significance at different stages following injury. We describe the epidemiology of early and late seizures following TBI, the significance of intracranial haemorrhage of different types in the risk of later epilepsy and the gaps in current understanding of risk factors contributing to the risk of post-traumatic epilepsy (PTE). The delay from injury to epilepsy presents an opportunity to understand the mechanisms underlying changes in the brain and how they may reveal potential targets for anti-epileptogenic therapy. We review existing treatments, both medical and surgical and conclude that current research is not tailored to differentiate between PTE and other forms of focal epilepsy. Finally, we review the increasing understanding of the frequency and significance of dissociative seizures following mild TBI.

## Introduction

Seizures were first described in relation to a “gaping wound of the head” in the Edwin Smith papyrus from Babylon, dated circa 1700 BC [1]. The Hippocratic physicians later recognised post-traumatic convulsions and their lateralisation opposite to the side of injury [2]. Through much of the next 2000 years, Galen’s philosophy of humours merged with spiritual explanations of epilepsy and it was not until the nineteenth century that understanding advanced to form the basis of current knowledge. In the UK, Gowers recognised the frequency of PTE and its male preponderance [3], which was followed shortly first epilepsy surgery of the modern era, conducted on a patient with PTE [4]. Holmes identified epilepsy as a cortical disease and used his extensive experience of traumatic brain injuries from WWI to describe a range of seizure types arising from injuries to different cortical regions [5]. Epilepsy surgery over the next decades was dominated by the Montreal Group who described the macroscopic and microscopic appearance of traumatic lesions associated with epilepsy in both humans and animals [6]. The extent of the risk was highlighted by Jennett [7] who set the investigative parameters for epidemiological studies which later investigators have followed in systematic attempts to delineate the risk factors for PTE [8, 9], but significant holes remain in our understanding. The better stratification of risk could potentially allow targeted treatment as epilepsy management starts to include treatments which may be anti-epileptogenic as well as anti-seizure. To date, the treatment of PTE has not been sufficiently separated from other forms of focal epilepsy to be able to give specific data. We also highlight the increasing recognition that seizures after mild traumatic brain injury are commonly dissociative rather than epileptic.

## Epidemiology

Over 2% of the population attend emergency departments in England and Wales each year with a head injury [10]. Between one-third and one half are children and one-fifth have a skull fracture or evidence of traumatic brain injury (TBI). Approximately 15% will require admission to hospital. Seizures may be almost instantaneous “concussive” episodes; early seizures within 7 days of injury and late epileptic seizures, beyond 7 days from injury.

Concussive seizures were originally described in Australian football players [11]. They are convulsive and occur within seconds of impact. Their semiology differs from tonic–clonic seizures and is postulated to contain elements of primitive reflexes [12]. They are believed to carry no risk of later epilepsy.

Early seizures were seen in 4.5% of a cohort of 1000 unselected patients with head injury in Oxford [7] and were associated with skull fracture or intracranial haemorrhage. Lee and Lui found that early seizures occur after 2.4% of 4232 head injuries, defined as mild, on the basis of clinical criteria Glasgow Coma Score (GCS), but imaging in a significant proportion subsequently showed significant haemorrhage, putting patients into a more severe category [13]. Over 80% of seizures were tonic–clonic rather than focal. A recent, large study [14] has expanded knowledge of the frequency of early seizures. They identified risk factors in a 2 year cohort of all admissions with TBI amongst patients over age 18 years from the National Trauma Data Bank of the USA. Of 360,863 patients admitted, only 1559 (0.4%) suffered early seizures. This is relatively few compared to other studies but even this cohort had a mortality of 7%, reflecting an inherently more severe group than the totality of TBI patients, many of whom are never admitted to hospital. There was an over-representation of Afro-Caribbean patients 20% v 12% for the population. A previous history of alcohol abuse was seen in 25% of those with early seizures versus 11% of those without, but intoxication at the time of admission was significantly less common 13% v 24%. Common comorbidities were all more frequent in those with seizures: smoking, obesity, hypertension or vascular disease. Risk of seizure was 0.38% of 273,327 with mild TBI, defined as Glasgow coma score (GCS) 13–15; 0.8% of 15,467 with moderate TBI (GCS 9–12) and 0.5% of 54,545 with severe TBI (GCS < 9). That moderate TBI was much less common than severe TBI, perhaps raises questions around severity definitions. Subarachnoid and subdural haemorrhage were associated with an increased risk but epidural and intraparenchymal haemorrhage were not. The vast majority of intraparenchymal haemorrhages were contusions, which seem to carry a low risk, whereas large intraparenchymal haemorrhages carry a much higher risk. Early seizures were associated with longer stay in intensive care, longer overall admissions a higher chance of discharge to a nursing facility rather than to home but no significant difference in mortality.

Studies have also looked at subclinical seizures, manifest on EEG monitoring. For example, in a study of 94 patients labelled as with moderate to severe TBI, Vespa found 21 suffered seizures; five tonic–clonic; four with subtle motor phenomena and the others purely electrographic. All six patients in status died, including one in whom there were no motor manifestations; four of these had a hypoxic insult superimposed on TBI [15]. Arndt et al. found that of children with TBI, subclinical seizures occurred in 16.1% of children undergoing monitoring and in 6.8% this was the only epileptic manifestation. Subclinical status epilepticus was reported in 13.8% and any epileptic manifestation, whether clinical or subclinical was associated with a poorer outcome from TBI in this cohort [16]. A consensus statement has recommended continuous EEG monitoring for a patients with a range of cerebral insults, including moderate to severe TBI, in whom there is a clinical suspicion of seizures, suggested by paroxysmal events of uncertain nature, altered or fluctuating mental state or anaesthesia and paralysis which may mask seizures in a clinical context, where there is significant risk [17]. This counsel of excellence is difficult to achieve in many centres and truly large-scale studies are lacking.

In an epilepsy population, approximately 5% of incident cases and approximately 20% of prevalent cases may be due to previous TBI [18–20]. The overall incidence of epilepsy is lowest in adults from 25 to 65, having emerged from childhood and adolescence without epilepsy due to genetic, developmental or perinatal causes and not yet suffering high rates of cerebrovascular or degenerative disease. But this group, especially males, have a significant risk of cerebral trauma and on a background of low risk from other causes, proportionately, they have the highest incident risk of PTE. However, any given individual’s risk of developing seizures after similar trauma may in fact be higher in the very young or the elderly [21].

Those who have suffered penetrating brain injuries carry the highest risk; for example just over half of Vietnam veterans with such injuries develop epilepsy [22] but these represent a small proportion of TBI in most societies. There have been a large number of studies of patients admitted to trauma units with TBI. Inevitably these studies are skewed to those with more severe TBI and the cohorts carry a greater risk. There have been three larger population-based studies, which have looked at injuries ranging from mild to severe [8, 9, 21] which have been compared, along with smaller studies in a meta-analysis [23]. Annegers divided patients’ TBI into mild, with less than 30 min post-traumatic amnesia (PTA) or loss of consciousness (LOC); moderate with 30 min–24 h PTA or LOC and severe with longer PTA or LOC, skull fracture or CT evidence of intracranial injury. The mild group carried no increased risk of seizures; the moderate group approximately 0.1% annual risk for 20 years and the severe group 5% in the first year, a further 5% by 5 years and 17% cumulative risk over 20 years from injury. Christensen’s study looked at children and young adults over 10 years after injury and divided patients into those with mild injury and severe injury and used a different analysis. Their mild group overlapped the Annegers classification of moderate and gave a similar risk, which was highest in the first year and their severe group also carried a similar risk to Annegers’ severe group. The categorisation in these studies has identified a step-shift in risk from mild or moderate cases to severe cases with high risk and begs the question whether there are identifiable biomarkers which might increase the granularity of risk prediction allowing prediction of risk intermediate between those categories. The Mayo clinic is a widely used severity classification, which combines different criteria in TBI severity assessment for the purpose of acute management and rehabilitation rather than epilepsy risk [24].

Yeh et al. used ICD-9-50 definition of concussion to classify a mild group of TBI, which will again have included patients in both Annegers’ mild and moderate groups and they defined a further skull fracture group and a severe TBI group on the basis imaging evidence of intracranial haemorrhage. They compared nearly 20,000 patients attending emergency departments from 2000 to 2003 with a head injury to more than 500,000 attending for other reasons and used a Cox proportional hazards model to identify different risk factors for developing epilepsy by 2008. The broad headlines were that epilepsy developed in 0.29% of controls; 1.1% of those with mild TBI; 4.8% of those with skull fractures and 2.2% of those with brain injury. Contusions represented 59% of the 2528 severe injuries and carried a risk of 1.6% of epilepsy, compared with 4.8% for subarachnoid haemorrhage; 6.3% for extradural haemorrhage; 7.8% for subdural haemorrhage; 10.2% for intracerebral haemorrhage and 13.9%, where there was more than one kind of haemorrhage. Most likely, some of the subtle contusions that were seen on imaging would not have been detected in the case ascertainment of the Annegers study from an earlier era of imaging, and would have fallen into their mild or moderate categories. These data suggest that small contusions should not be considered in the same risk category as the other forms of haemorrhage or skull fracture and this study helpfully starts to break down the risk by lesion type.

As well as the nature of the injury, additional risk factors for the development of PTE have been identified including a prior history of alcohol abuse [23]. An aging brain may increase seizure risk [21] as may a family history of epilepsy; a genetic predisposition on which TBI is superimposed [9]. Early post-traumatic seizures, within the first week, are also associated with a significantly greater chance of later epilepsy [7, 9]. The brunt of cerebral trauma is well known to be carried by the frontal and anterior temporal lobes, related to bony structures of the skull but lesion location has been inconsistently associated with seizure risk, with studies pointing to different parts of the brain as carrying the highest risk [22, 25].

## Prevention of seizures and epilepsy

Where patients may be at high risk of developing epilepsy, it is tempting to consider medication to prevent seizures. A Cochrane review of prophylactic antiepileptic drugs after TBI concluded that studies were of poor quality. They found that treatment (phenytoin or carbamazepine) may reduce the incidence of early post-traumatic seizures but has no impact on late seizures or on mortality after TBI [26]. Valproate and levetiracetam were also compared to phenytoin and no benefit was found. The Brain Trauma Foundation Guideline does not make a recommendation [27]. Prophylaxis for those who have not had seizures cannot be recommended without better classification of risk. However, a discrete event, bringing a patient to medical attention with a significant later epilepsy risk, is a relatively unusual situation in clinical epilepsy and presents an archetype to consider anti-epileptogenesis in humans.

Following injury there are a number of processes, including necrosis, microhaemorrhage, axonal injury, apoptosis, demyelination, microgliosis, inflammation and oxidative stress and later phases of neurodegeneration, regeneration, revascularisation and remodelling which may contribute to the circuit changes resulting in later epilepsy [28]. The challenge is to identify biomarkers for these processes and target interventions. Imaging markers such as gradient echo MRI for blood products or diffusion tensor MRI for pathway remodelling, evidence of thalamic or hippocampal damage may help to stratify risk [29]. Genetic markers help stratification of the interaction between acquired and constitutional risk. There is increasing evidence for degenerative processes such as Tau accumulation in post-traumatic epileptogenesis [30] or inflammatory changes, for example with IL-1ß levels raised in the CSF of patients who later developed epilepsy [31]. Neurophysiological changes such as changes in sleep spindles or EEG high frequency oscillations identified in animal studies may also translate to human epileptogensis but studies suffer from the bias of being selectively undertaken in those at the highest risk [32]. In animal models, treatments reducing accumulation of Tau protein; targeting iron accumulation; targeting inflammatory pathways and calcium channels involved in apoptosis have all shown some success in preventing development of PTE. None is yet of proven benefit in humans [33]. Rapamycin, an inhibitor of the mTOR pathway has also shown evidence of anti-epileptogenesis in animals and has demonstrated efficacy in lesion reduction and seizure prevention in humans with tuberous sclerosis [34, 35]. It is unlikely to be used widely but represents a welcome proof of principle of anti-epileptogenesis in a field, where there have been many false starts.

## Treatment of seizures associated with traumatic brain injury

There is one randomised trial of phenytoin v placebo in the treatment of early seizures in association with TBI [36]. This demonstrated a reduction in early seizures but in the continuation phase, no reduction in the later development of epilepsy. Levetiracetam has no benefit compared to phenytoin [37]. The Brain Trauma Foundation Guideline supports the use of phenytoin for early seizures [27]. A recent survey of clinicians in the UK and Ireland suggest that around half do not use prophylactic anti-epileptic drugs (AED) but 38% use them for a month and 90% were unsure of the correct duration of treatment. Levetiracetam was the favoured drug [38]. Patients developing seizures are commonly treated with medications which are used in acute units in status epilepticus, as these can achieve therapeutic doses. Most commonly, these are phenytoin or levetiracetam but valproate has also been used moderately frequently in status epilepticus. Recent evidence suggests that there is little to choose between these medications in this context [39, 40].

Patients with PTE will have nearly all suffered significant traumatic brain injury and often have cognitive, executive, behavioural, emotional and sometimes physical consequences of their injuries as well as epilepsy. There is a significant associated psychosocial morbidity and a mortality as well as associated drug use or alcoholism which profoundly affect quality of life but also have an impact on epilepsy treatment [41–44]. These aspects need to be considered in management but are beyond the remit of this article. Equally, late mortality may be significantly higher in those with epilepsy than in those with otherwise similar injuries and no epilepsy [45].

In terms of epilepsy classification, PTE is a syndrome with generalised and focal seizures and a structural cause [46], previously known as focal, remote symptomatic epilepsy. Clinical trials of AED in this group do not differentiate between aetiologies and there are no trials of medication specifically for patients with PTE. The considerations are, therefore, to achieve seizure control, if possible and avoid specific comorbidities that may be associated with TBI. Especially if behavioural or mood issues are of particular concern, then the authors’ first choice for long term treatment would be lamotrigine, carbamazepine, oxcarbazepine or lacosamide. Lamotrigine may be associated with headaches and sleep disturbance, which may affect those with TBI and carbamazepine and oxcarbazepine may exacerbate hyponatraemia in those with inappropriate ADH secretion. Whilst irritability is more common with levetiracetam it does not preclude its use with appropriate monitoring and brivaracetam may be useful in those who suffer this complication but whose epilepsy responds well to levetiracetam.

Approximately one-third of epilepsy will prove refractory to medication and it is not clear if there is any systematic difference in post-traumatic cases compared to other causes. In this group epilepsy surgery may be considered. Since Horsley [4] it has been known that this can be a successful treatment for PTE. Modern studies confirm that it may be successful in those appropriately selected, where the injury is either unifocal, or if multifocal, that investigation confirms that only one region is epileptogenic and where the patient is able to cope with the neuropsychological consequences of resection [47]. Another, larger study also had good outcomes but probably included a significant number of non-traumatic cases, since their definition of trauma included all injuries with PTA of > 30 min and 44% had mesial temporal sclerosis, not generally associated with traumatic injury [48] Vagus nerve stimulation has been suggested to work better for PTE with 73% seizure reduction at 24 months compared to 57% for non-TBI related epilepsy [49].

## Head injury and dissociative seizures

Clinicians may be tempted to assume that seizures occurring following head injury are epileptic. However, TBI is also associated with dissociative seizures (previously called psychogenic non-epileptic seizures). US epilepsy centres identified this association in the 1990’s, and a recent review of 17 subsequent studies corroborated this link in adult and paediatric populations [50–52]. Dissociative seizures superficially resemble epileptic seizures, and 1 in 5 patients referred to epilepsy clinics with a prior diagnosis of refractory PTE actually have dissociative seizures [53, 54]. Where an injury has been mild and the a priori risk of epilepsy is very low, post-traumatic seizures are more likely to be dissociative than epileptic [50, 51]. Most reports point to onset of nearly all dissociative seizures (81–89%) within a year of TBI, whereas epilepsy risk declines more gradually in the years following trauma [8]. Patients with dissociative seizures also show a lack of response to anti-epileptic drugs (AEDs), which could be perceived as a ‘red flag’ for their diagnosis [55]. AEDs have additionally been observed to worsen outcomes in some patients with dissociative seizures [55]. Association with post-traumatic stress disorder (PTSD) and other psychiatric comorbidities further delineates post-traumatic dissociative seizures from epileptic seizures. For example, 57% of US veterans with post-traumatic dissociative seizures also have PTSD, compared with an overall PTSD prevalence of less than 10% observed in another cohort of veterans [57, 58]. A higher rate of other psychiatric comorbidities than in PTE, significantly increases illness burden for many patients.

Overall, significantly more women than men (3:1) are affected by dissociative seizures, compared to a male preponderance for epileptic seizures [59]. This may be due to greater female vulnerability to any number of predisposing factors [60]. The majority of patients developing post-traumatic dissociative seizures are female [51]. Other recognised associations of dissociative seizures, such as physical and sexual abuse, may need to be sought for a holistic assessment [61].

Psychological therapies are the mainstay of dissociative seizure management, giving significant symptomatic improvement but relatively few patients with dissociative seizures can expect to become seizure-free [62]. Diagnostic delay is greater than for epilepsy and is a factor which may adversely affect prognosis [56]. Increased awareness of the distinguishing features of post-traumatic dissociative seizures may help to tease these cases apart from their epileptic counterparts enabling appropriate management for the best chance of seizure reduction and improved quality of life.

## Summary

Seizures are a common complication of moderate to severe head injury, which may arise over many years. Intracerebral haemorrhage but not small contusions, carry a high risk and further work needs to be done to stratify cases. We are starting to understand some of the mechanisms underlying epileptogenesis, but this has not yet translated into preventative treatments. Our current information is not sufficiently granular to separate treatment of PTE from other forms of focal epilepsy but medication, respective surgery and VNS may all be useful. Dissociative seizures are common and require a high index of suspicion after mild injury.
